# Exploring the frontier: nonlinear optics in low dimensional materials

**DOI:** 10.1515/nanoph-2024-0652

**Published:** 2025-03-10

**Authors:** Mohammad A. Adeshina, Hyunmin Kim

**Affiliations:** Division of Biomedical Technology, Daegu Gyeongbuk Institute of Science and Technology (DGIST), Daegu 42988, Republic of Korea; Department of Interdisciplinary Engineering, Daegu Gyeongbuk Institute of Science and Technology (DGIST), Daegu 42988, Republic of Korea

**Keywords:** nonlinear optics, low dimensional materials, optical wave mixing, biological imaging

## Abstract

Nonlinear optics, the study of intense light–matter interactions, traditionally uses bulk materials like LiNbO_3_ for device fabrication. However, these materials face challenges such as limited nonlinear susceptibility, large dimensions, and phase matching issues, limiting compact and integrated devices. Recent research has illuminated that a variety of low-dimensional materials exhibit markedly stronger nonlinear optical responses than their bulk counterparts. This has made nonlinear optics in low-dimensional materials a dynamic area of study, allowing for rapid light–matter interactions and advancing nonlinear nanophotonic and optoelectronic applications. These applications span diverse areas, from wavelength conversion and the generation of ultrashort laser pulses to advancements in quantum photonics and integrated photonic technologies. This review covers two-dimensional materials such as graphene and transition metal dichalcogenides to one-dimensional forms like carbon nanotubes and nanowires, and further to zero-dimensional structures including nanoparticles and quantum dots. By providing a comprehensive overview of the current state of non-linear optics in the context of low-dimensional materials, this review not only encapsulates the existing knowledge base but also charts a course for future explorations in this rapidly progressing domain.

## 1 Introduction

Nonlinear optics is a field of study that explores the interaction of light with materials under high-intensity light fields, leading to phenomena that do not occur under low-intensity illumination. The optical properties of a material can change in response to the intensity of the light, resulting in the generation of new frequencies or changes in the speed of light [1], [2]. Low-dimensional materials (LDMs), including two-dimensional (2D), one-dimensional (1D), and zero-dimensional (0D) materials, have emerged as promising platforms for nonlinear optics due to their unique properties. Since the groundbreaking discovery of graphene in 2004 [3], [4], [5], there has been an exponential increase in the study and interest in low dimensional materials. These materials, which include Graphene [6], [7], [8], [9], [10], Transition Metal Dichalcogenides (TMDs) [11], [12], [13], [14], [15], [16], Black Phosphorus (BP) [17], [18], [19], [20], [21], topological insulators [22], [23], [24], MXenes [25], Nanotubes [26], Nanowires [27], Nanoparticles and Quantum dots [28], [29], have been found to exhibit atomically thin, layered structures and exceptional physical phenomena [30]. The rise of nonlinear photonics in LDMs began around 2009 and has since become a significant research direction [31]. The reduced dimensionality of these materials, along with their unique electronic and optical properties, offers new possibilities for manipulating light at the nanoscale [30], [32].

For instance, 2D materials, such as graphene and TMDs, have shown exceptionally strong nonlinear optical responses, benefiting from their reduced dimensionality, relaxed phase-matching requirements, and enhanced light–matter interaction [33]. One-dimensional materials like carbon nanotubes and nanowires, and zero-dimensional materials like quantum dots and nanoparticles, also exhibit intriguing nonlinear optical properties [32]. Moreover, the advent of novel nano-/microstructures, such as metasurfaces and heterostructures, has further expanded the scope of nonlinear optics in low-dimensional materials. Low-dimensional heterostructures, including 2D-2D (graphene-TMD), 2D-1D (graphene-carbon nanotube), and 2D-0D (TMD-quantum dot) systems, offer remarkable opportunities for enhanced nonlinear optical phenomena. Interlayer coupling in heterostructures enables second-harmonic generation (SHG) enhancements, while twisted bilayers create moiré superlattices that exhibit emergent nonlinear optical behaviors, such as symmetry-broken SHG and third-harmonic generation (THG) [33]. These structures provide tunable platforms for hybrid photonic devices. Nonlocal metasurfaces, which respond to light collectively, have shown promise in enhancing and controlling nonlinear optical phenomena [34]. These structures provide additional degrees of freedom in modulating the physical properties of the materials, leading to fascinating nonlinear optical phenomena [33].

The field of nonlinear optics in low-dimensional materials holds significant importance and relevance in both scientific research and practical applications. The unique properties of low-dimensional materials, such as their reduced dimensionality and enhanced light–matter interaction, have opened up new avenues for the study and manipulation of light at the nanoscale. The study of nonlinear optics in these materials is not only of fundamental scientific interest but also has significant implications for technological advancements [35]. The nonlinear optical responses of these materials have been utilized in a wide range of applications, including laser technology, ultrafast photonics, integrated photonics devices, nonlinear light control, and bio-imaging [36], [37], [38], [39], [40]. In laser technology, for instance, the strong nonlinear optical responses of low-dimensional materials can be harnessed to develop more efficient and compact lasers. In the field of ultrafast photonics, these materials can be used to generate and manipulate ultrafast light pulses, which are crucial for high-speed optical communications [32]. Moreover, the field of nonlinear optics in low-dimensional materials is closely related to the development of integrated photonics devices. These devices, which integrate multiple optical functions onto a single chip, are expected to revolutionize information technology by enabling faster and more energy-efficient data processing and communication [41], [42]. The relevance of this field extends beyond these applications. The study of nonlinear optics in low-dimensional materials can also provide valuable insights into the fundamental properties of these materials, contributing to the broader understanding of light–matter interactions at the nanoscale [43], [44].

**Figure 1:**
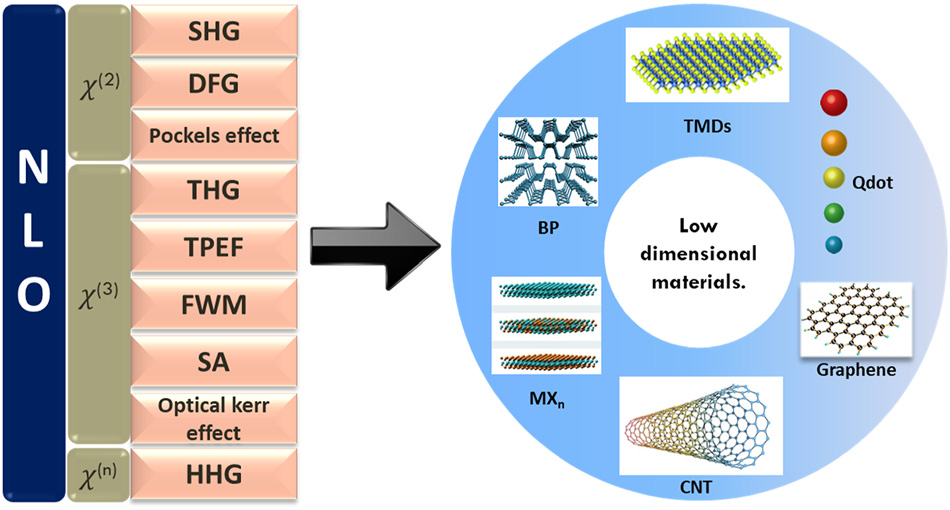
Schematic representation of prominent non-linear optical processes and common low-dimensional materials.

**Figure 2:**
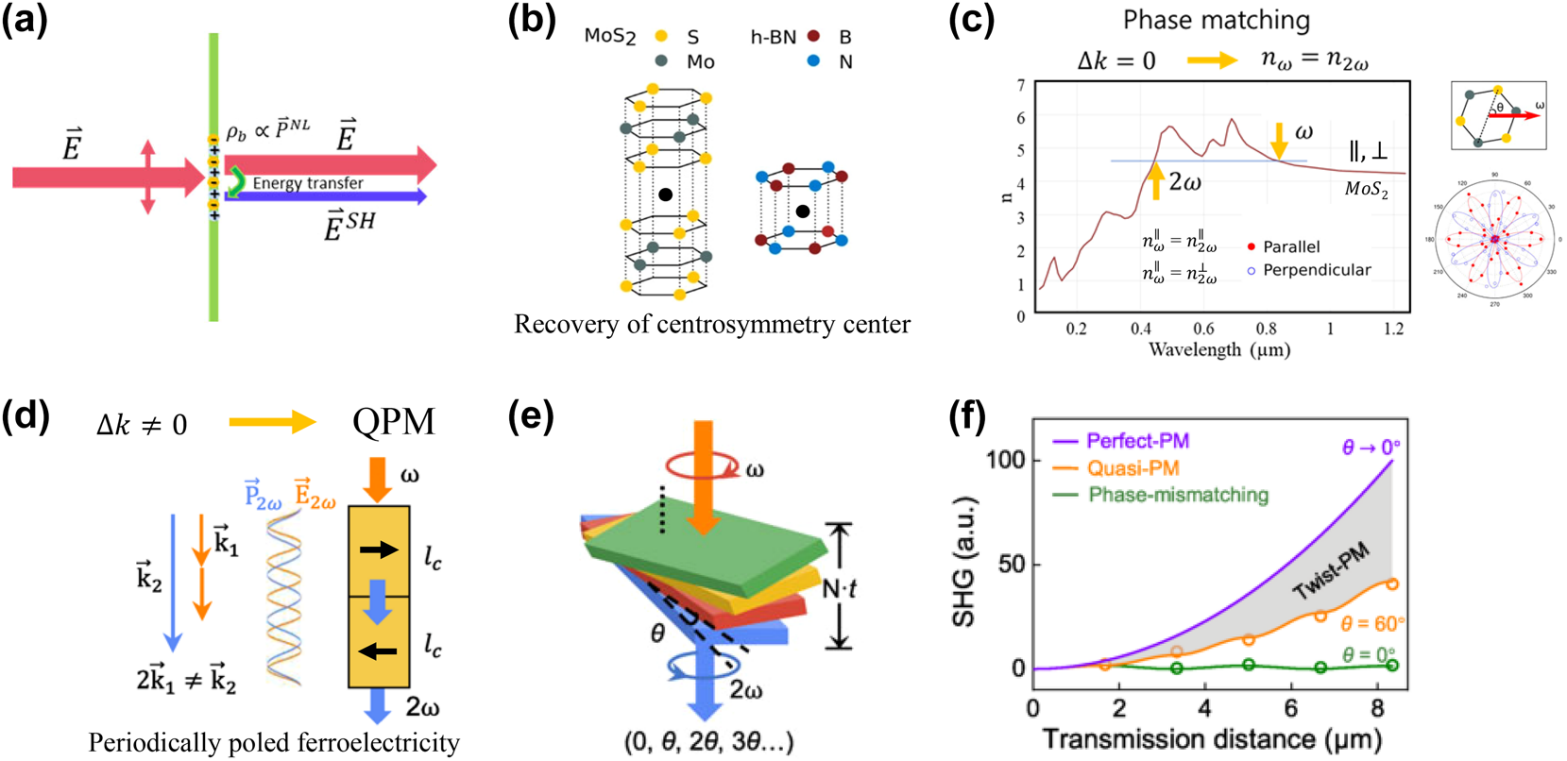
Nonlinear optical processes and properties in low-dimensional materials. (a) Schematic of the second harmonic generation (SHG) process, illustrating energy transfer and (b) recovery of centrocymmetry and [45] adapted with permission from Copyright 2013 American Chemical society (c) refractive index matching in MoS_2_ for efficient SHG. QPM (quasi phase matching) condition achieved by (d) a periodically poled crystal and (e) a twisted stacking of the low dimensional structure. (f) SHG efficiency comparison among various phase matching conditions [48]. Reprinted with permission Copyright 2023 American Physical Society.

Figure 1 shows an illustration of commonly used low dimensional materials. One of the most important concepts in the synergy of NLO and LDM is Phase matching. This is crucial for efficient energy transfer between light frequencies (Figure 2(a) and (b)) [45]. When the phase velocities of the waves align, it leads to constructive interference and conversion of optical frequencies. Achieving phase matching in low-dimensional materials like atomically thin layered 2D materials offers unique opportunities (Figure 2(c)) [46], [47]. Interlayer twist angles in 2D materials have been found to induce a nonlinear geometric phase, enabling the overcoming of phase mismatch and introducing a novel “twist-phase-matching” regime as shown in Figure 2(d)–(f). Traditional QPM, achieved through the insertion of the dimension-controlled (*l*_*c*_: coherent length) alternating polarization in bulk ferroelectric materials (Figure 2(d)), is contrasted with a novel twist-based phase matching technique in Figure 2(e). By introducing an interlayer twist angle in materials like MoS_2_ and h-BN, phase mismatch is compensated, leading to enhanced SHG. Figure 2(f) demonstrates the dependence of SHG efficiency on the twist angle, with a peak at 60°, and confirms the superior performance of this approach over conventional QPM, especially for miniaturizing nonlinear optical devices [48]. These advances hold the most important factor for SHG and other phenomena actualization.

Furthermore, the advent of low-dimensional materials has precipitated a series of pivotal breakthroughs, attributable to their distinct electronic and optical properties that diverge markedly from their bulk counterparts. Graphene, with its linear dispersion of Dirac electrons, underpins a broad spectrum of nonlinear optical responses, thereby advancing the field of ultrafast photonics through its employment in saturable absorbers for mode-locking applications [49], critical for the generation of femtosecond laser pulses. The monolayer variants of TMDs, such as MoS_2_ and WS_2_, have demonstrated pronounced SHG owing to their inherent non-centrosymmetric lattice configurations, facilitating novel SHG microscopy techniques, particularly for biological imaging applications [50]. Furthermore, topological insulators like Bi_2_Se_3_ have been the subject of intense study due to their high third-order nonlinear susceptibilities, which hold promise for high-speed, all-optical modulation within photonic circuits [51], [52]. BP, characterized by its thickness-dependent bandgap, has emerged as a versatile material for broadband tunability applications, spanning photodetectors to optical switches [53]. Carbon nanotubes, with their pronounced third-order nonlinear optical response and rapid temporal reaction, alongside semiconductor nanowires, are driving innovations in compact, efficient photonic devices due to their unique quantum confinement [54]. Quantum dots, with their size-tunable electronic properties, excel in multi-photon absorption and frequency up-conversion, which are quintessential for applications ranging from biological sensing to the realm of quantum computing [55], [56], [57]. Lastly, MXenes, a burgeoning class of 2D materials, exhibit adjustable surface chemistry and plasmonic characteristics that are seminal for amplifying nonlinear optical effects [58]. Their deployment in applications ranging from optical switching to photonic circuit fabrication heralds a new era of integrated optical and electronic systems, promising a future replete with advancements in telecommunications, quantum information, and sensory technologies. This synergistic integration of LDMs is anticipated to be a cornerstone of forthcoming optical computing advancements, signaling a rich landscape of technological innovation. Despite the significant progress made in this field, further theoretical and experimental investigations are needed to fully understand and exploit the nonlinear optical properties of low-dimensional materials. The complex nature of NLOs, in LDMs systems, requires an interdisciplinary approach and innovative thinking. As research in this field continues to advance, it is anticipated that new insights will be gained into the underlying mechanisms of nonlinear optics in low-dimensional materials, leading to the development of more sophisticated applications [59], [60], [61], [62].

The primary objective of this review paper is to provide a comprehensive overview of the recent progress in the field of NLOs in LDMs. This includes a detailed examination of the theoretical models, experimental techniques, and the specific low-dimensional materials that have shown promising nonlinear optical properties. The survey aims to highlight the significant advancements in the understanding of nonlinear optical phenomena in these materials, and how these insights have been applied. In addition to summarizing the current state of the field, this review also aims to identify the challenges and opportunities that lie ahead. This includes discussing the limitations of current theoretical models and experimental techniques, as well as the potential for new materials and structures to enhance nonlinear optical responses. By providing a comprehensive overview of the recent progress in this field, this review aims to serve as a valuable resource for researchers and practitioners in the field of nonlinear optics, materials science, photonics, and related disciplines. It is hoped that this review will stimulate further research and innovation in the field of nonlinear optics in low-dimensional materials.

## 2 Band structures and optical properties of low dimensional materials

**Figure 3:**
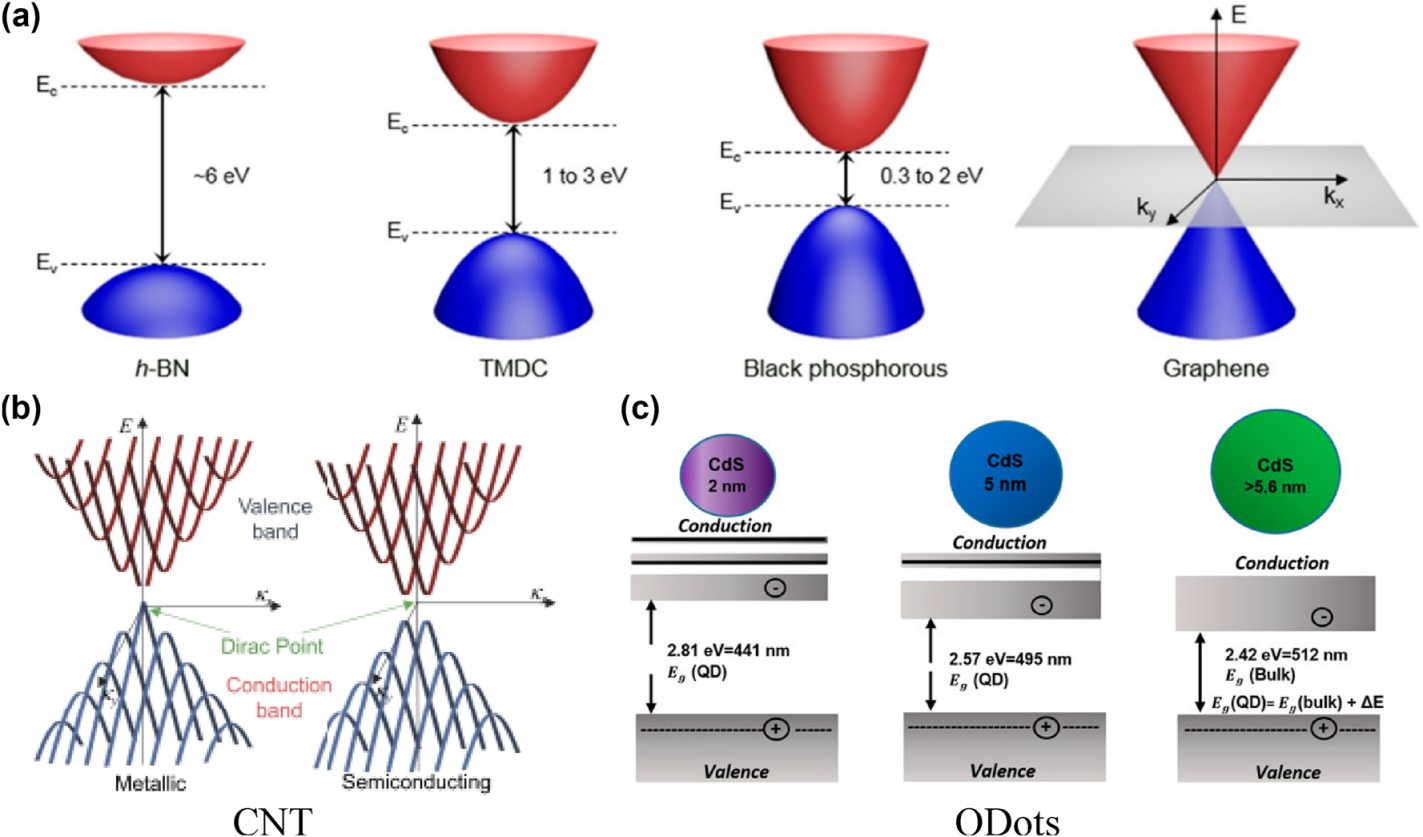
Electronic band structures of varied low-dimensional materials; (a) presents 2D materials [73], Reproduced with permission under Creative Commons license. (b) depicts 1D materials, and [74] Reproduced with permission under Creative Commons license. (c) illustrates 0D quantum dots [75]. Reprinted with permission Copyright 2010 Wiley.

**Figure 4:**
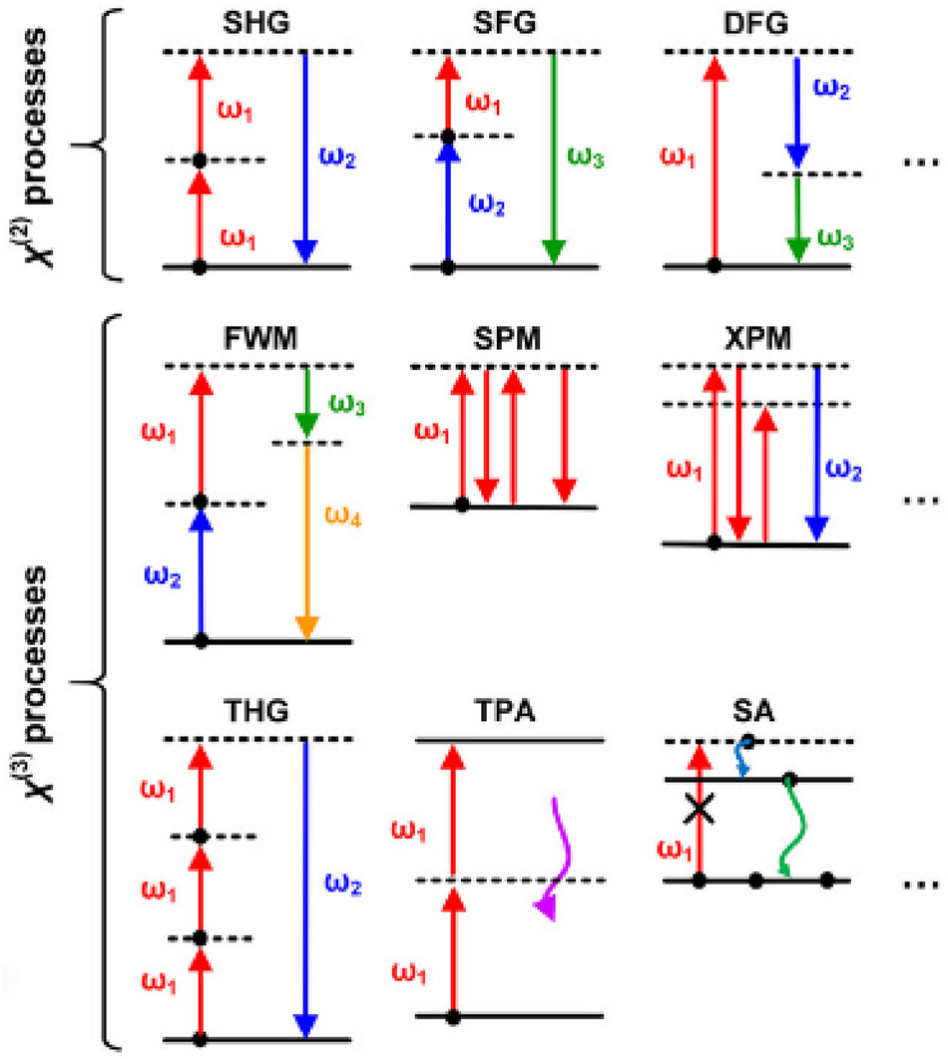
Schematic representation of various nonlinear optical phenomena categorized by second-order (*χ* ^(2)^) and third-order (*χ* ^(3)^) nonlinear optical processes, showcasing SHG (second-harmonic generation), SFG (sum frequency generation), DFG (difference frequency generation), FWM (four-wave mixing), SPM (self-phase modulation), XPM (cross-phase modulation), THG (third-harmonic generation), TPA (two-photon absorption), and SA (saturable absorption) [64]. Reproduced with permission under Creative Commons license.

The exploration and understanding of band structures and optical properties in LDMs have been instrumental in propelling advancements in the field of NLOs. Graphene, notable for its zero-bandgap and linear dispersion near the Brillouin zone’s *K* points, showcases a spectrum of remarkable optical attributes, including extensive absorption and pronounced third-order nonlinear susceptibility [63]. These characteristics have established graphene as a formidable player in ultrafast photonics, particularly in the development of mode-locked lasers for generating rapid optical pulses [64]. Similarly, TMDs, such as MoS_2_ and WS_2_, reveal direct bandgaps in their monolayer structures, enabling significant nonlinear responses like potent SHG and two-photon absorption, essential for applications in nonlinear imaging and photodetection [65]. Quantum dots, exhibiting size-dependent bandgaps due to quantum confinement, lead to discrete energy levels and size-tailored nonlinear optical properties, leveraged in diverse applications from bioimaging to quantum information processing [55], [66], [67]. Moreover, semiconductor nanowires, by virtue of their one-dimensional nature, demonstrate notable quantum confinement effects, translating into unique electronic and optical properties, particularly valuable for photonic circuits in frequency mixing and optical switching [68], [69], [70]. Figure 3(a) shows the band structures of an array of LDMs, including hexagonal boron nitride (h-BN), TMDs, BP, and graphene, each epitomizing the characteristics of two-dimensional (2D) materials. The significant bandgap in h-BN and the adjustable bandgaps in TMDs and BP suggest their potential for high harmonic generation and frequency mixing [53], [71], [72], [73]. The figure also contrasts the metallic and semiconducting behaviors in one-dimensional and QDots materials (Figure 3(b) and (c)), highlighting how the presence or absence of a band gap at the Dirac point can impact conductivity and, consequently, nonlinear optical responses [74], [75]. Collectively, the band structures and optical properties of these low-dimensional materials underscore their versatility and broad applicability, significantly enhancing the scope of nonlinear optical applications and contributing to the evolution of advanced photonic and optoelectronic devices. In the advanced field of photonics, phase matching plays a pivotal role in a spectrum of NLO phenomena. These phenomena are illustrated in Figure 4, categorized into second-order (*χ* ^(2)^) and third-order (*χ* ^(3)^) processes. SHG and THG are prime examples where photons coalesce within a nonlinear medium to form new photons with frequencies that are multiples of the original [64]. SHG is notably evident in layered TMDs due to their non-centrosymmetric crystal structures [76], while THG finds its efficacy in thin films and quantum wells for frequency upconversion [77]. Sum Frequency Generation (SFG) and Difference Frequency Generation (DFG) demonstrate the addition or subtraction of photon frequencies, processes influenced by the enhanced electric fields in materials like graphene [78], [79]. Additionally, Four-Wave Mixing (FWM), Self-Phase Modulation (SPM), and Cross-Phase Modulation (XPM) are significant third-order processes observed in materials with high third-order susceptibility [80], [81], crucial for ultrafast optoelectronic applications. Two-Photon Absorption (TPA) is particularly pronounced in quantum dots and plays a key role in the design of optical limiting devices [82], [83]. Saturable Absorption (SA), where absorption decreases with increasing light intensity, is vital in the mode-locking of lasers, a mechanism effectively employed in carbon nanotubes and other low-dimensional materials for the generation of ultrafast laser pulses [84], [85], [86], [87]. These NLO phenomena, through their diverse applications in high-resolution imaging, sensing, and information processing, underscore the significant role of phase matching in LDM in advancing photonics, facilitating the creation of sophisticated optical systems and devices that extend the frontiers of scientific research and technological innovation.

## 3 Fabrication technology for integrating 2D materials

**Figure 5:**
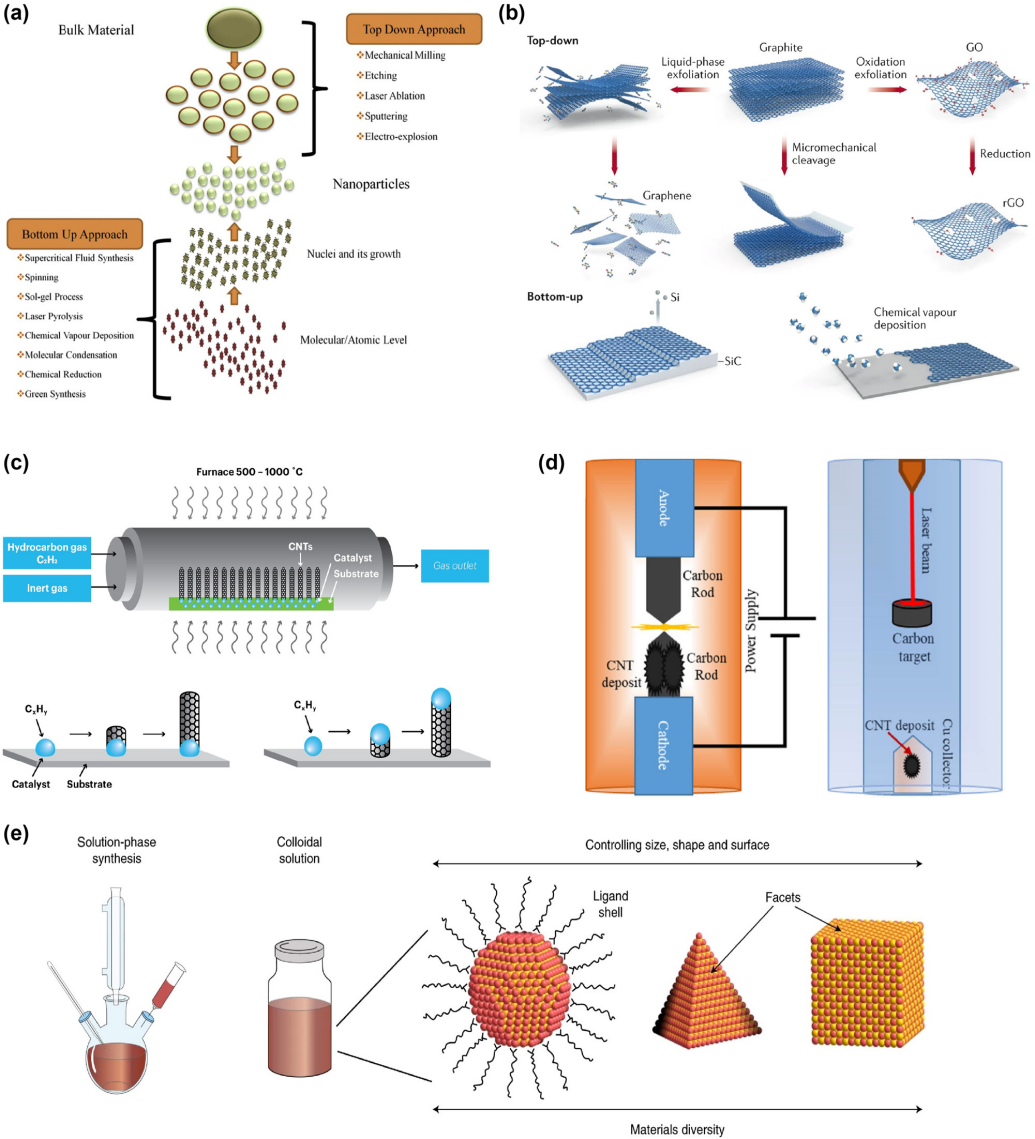
Synthesis techniques for low-dimensional materials, illustrating top-down and bottom-up approaches for zero-, one-, and two-dimensional structures. (a) Overview of synthesis techniques for low-dimensional materials via Top-down and Bottom-up approaches [88]. Reproduced with permission Copyright 2019 Elsevier. (b) A compilation of synthesis methods for two-dimensional graphene materials, depicting established methods for synthesizing low-dimensional structures [89]. Reproduced with permission Copyright  2017 Macmillan Publishers Limited. (c) Chemical vapor deposition (CVD) for the production of one-dimensional nanotubes [90]. Reproduced with permission Copyright 2021 Tuball. (d) Arc discharge technique utilized in generating one-dimensional nanostructures [91]. Reproduced with permission Copyright 2019 Elsevier. (e) Solution-based synthesis process for the creation of zero-dimensional quantum dots (Qdots) [92]. Reproduced with permission Copyright 2021 Springer Nature Limited.

Generally, the synthesis of LDMs is divided into two primary methodologies: Top-Down and Bottom-Up approaches, each distinguished by unique processes and applications. The Top-Down approach, as shown in Figure 5(a), involves the deconstruction of bulk materials into nanoscale structures through various techniques [88].

This category includes mechanical milling for breaking down large pieces, etching and laser ablation for layer removal, Sputtering for atom ejection, and electro-explosion for material fragmentation. These techniques are characterized by their straightforwardness and scalability, making them suitable for large-scale production [93], [94], [95]. Conversely, the bottom-up approach focuses on assembling materials from the molecular or atomic level, promoting the nucleation and growth of nanostructures. This approach encompasses a range of techniques, from supercritical fluid synthesis and spinning methods to the sol–gel process, laser pyrolysis, and chemical vapor deposition (CVD) [96]. It also includes molecular condensation, chemical reduction, and green synthesis, an eco-friendly method utilizing biological entities. This approach is acclaimed for its precision and the ability to create complex structures with fewer defects, although it can be complex and cost-intensive for mass production [97]. The selection between these methodologies profoundly affects the structural and functional properties of the resultant LDMs, including graphene, TMDs, quantum dots, and nanowires. The strategic choice of the synthesis method hinges on the required material characteristics, scalability, cost-efficiency, and the intended application [98], [99].

Figure 5(b) shows the distinct fabrication technologies employed for the integration of two-dimensional materials, in this case for graphene, through both top-down and bottom-up approaches. In the Top-Down approach, the process begins with bulk graphite, which is subjected to liquid-phase exfoliation or oxidative exfoliation to yield graphene and graphene oxide (GO), respectively [89]. The former technique disperses and separates layers in a solvent, whereas the latter introduces oxygen functionalities into the graphite structure. GO can then be chemically reduced to produce reduced graphene oxide (rGO), a material closer to pristine graphene but with residual functional groups [100], [101], [102], [103]. Micromechanical cleavage, another top-down method, involves the physical separation of graphene layers using adhesive tapes, a technique known for producing high-quality but small-sized flakes [104]. The bottom-up approach, on the other hand, constructs graphene from the atomic level, often utilizing CVD on catalytic substrates such as silicon carbide (SiC). This method facilitates the arrangement of carbon atoms into a graphene lattice directly on the substrate, producing large-area, high-quality graphene sheets [105], [106].

When considering other two-dimensional materials such as h-BN, TMDs, BP, and MXn, the synthesis approaches must be adapted to their unique properties. For h-BN, CVD is often employed, similar to graphene, to produce layers with a similar structure but with insulating properties [107], [108]. TMDs, like MoS_2_ or WS_2_, can be synthesized through CVD, physical vapor deposition (PVD), or molecular beam epitaxy (MBE), allowing for controlled layer number and stacking [109], [110], [111]. BP can be exfoliated from its bulk form or synthesized through gas-phase reactions, where precision in maintaining its layered structure and protecting it from oxidation is key [112]. MXenes, derived from MAX phases, typically involve selective etching of the A layers in the bulk material to produce 2D carbides or nitrides [113], [114].

Each material presents unique challenges and opportunities in synthesis. For instance, h-BN requires maintaining a wide bandgap [115], while TMDs must retain their direct bandgap in single layers for electronic and photonic applications [116]. BP demands stability against environmental degradation [117], and MXenes require control over their surface terminations to modulate electrical conductivity [118].

The various fabrication technologies employed for the synthesis of one-dimensional (1D) and zero-dimensional (0D) materials, particularly carbon nanotubes (CNTs) and quantum dots, respectively are also discussed. Figure 5(c) illustrates the CVD method for CNTs, where a hydrocarbon gas is decomposed in the presence of a metal catalyst at high temperatures, resulting in the growth of CNTs on the substrate [90]. This bottom-up approach allows for the precise control of the nanotube’s characteristics by adjusting the catalyst, temperature, and gas composition. Figure 5(d) showcases alternative methods such as arc discharge and laser ablation for CNT synthesis, which can be considered top-down approaches as they start from bulk carbon sources and utilize physical forces to generate CNT structures [91]. These methods, while capable of producing high-quality nanotubes, often require subsequent purification steps [119]. For the synthesis of quantum dots, solution-phase synthesis is mostly used as depicted in Figure 5(e). A colloidal approach where precursors react in a solvent, leading to the nucleation and growth of quantum dots [92]. This bottom-up method provides the ability to tailor the size, shape, and surface properties of the quantum dots by carefully controlling the reaction conditions, such as temperature, solvent, and ligand chemistry [120].

Integrating such LDMs into devices necessitates a comprehensive understanding of the synthesis methods to ensure the materials’ properties are optimized for their intended applications. For instance, the diameter and chirality of CNTs, which are critical for electronic properties, are determined during the synthesis phase [121]. Similarly, the NLO properties of quantum dots, are a direct result of their size and surface chemistry, which are controlled during the colloidal synthesis process. These varied fabrication techniques highlight the nuanced complexity and flexibility offered by both top-down and bottom-up approaches in the development of LDMs.

## 4 Non-linear optical setups and responses of various LDM

### 4.1 Transition metal dichalcogenides (TMDs)

Transition Metal Dichalcogenides (TMDs), such as MoS_2_, WS_2_, MoSe_2_, and WSe_2_, have gained significant attention in nonlinear optics due to their distinct two-dimensional (2D) properties. These materials exhibit a variety of nonlinear phenomena, including SHG, FWM, and Third-Harmonic Generation (THG), primarily driven by their structural symmetry and unique bandgap configurations [122], [123], [124], [125].

**Figure 6:**
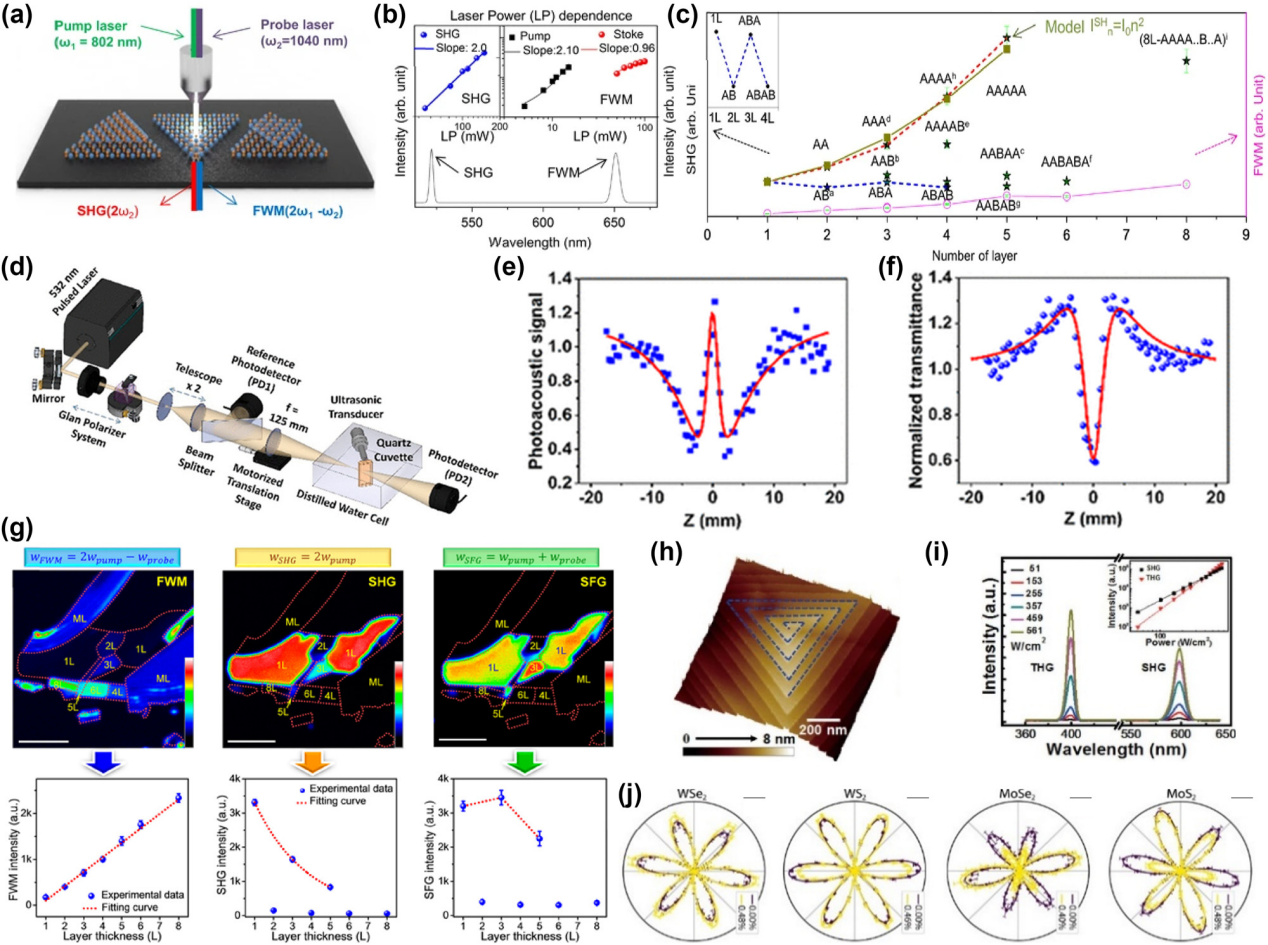
Nonlinear optical phenomena in transition metal dichalcogenides (TMDs), showcasing SHG, THG, and FWM signals and their dependence on laser power, thickness, and stacking. (a) Experimental setup with pump and probe lasers for generating second-order SHG and third-order FWM nonlinear optical signals (b) laser power dependence of SHG and FWM intensities [126]. Reproduced with permission under Creative Commons. (c) SHG and FWM signals from various layer thicknesses and stacking configurations showing the correlation between layer number and nonlinear optical response [126]. Reproduced with permission under Creative Commons license. (d) Experimental setup used for measuring simultaneously the open and closed aperture [127]. Reproduced with permission Copyright 2020 American Chemical Society. (e) Open aperture optical *Z*-scan signatures for different intensities (f) respective photoacoustic *Z*-scan signatures [128]. Reproduced with permission Copyright 2020 American Chemical Society. (g) Multiphoton nonlinear optical images of a mechanically exfoliated few-layer MoS_2_ flakes with different thicknesses and nonlinear optical signals of FWM, SHG, and SFG as functions of MoS_2_ layer thickness [129]. Reproduced with permission Copyrigh 2016 American Chemical Society. (h) AFM image of a spiral WS_2_ nanosheet, and the blue guidelines indicate the structures of the nanosheet (i) WS_2_ SHG and THG effects under different power densities. Inset shows the plot of the intensity of SHG and THG with increasing power density [130]. Reproduced with permission Copyright 2017 American Chemical Society. (j) Polarization dependent SHG measurements of MoS_2_, MoSe_2_, WS_2_, and WSe_2_, under lowest and highest applied strain. Levels [131]. Reproduced with permission under Creative Commons license.

In multi-stacked MoS_2_ crystals, the stacking order plays a crucial role in determining the nonlinear optical behavior. Recent studies have revealed that MoS_2_ stacked in AA(A…) configurations exhibits significantly enhanced SHG intensity compared to its natural 2H-phase counterpart with AB(A…) stacking. This enhancement is due to the broken inversion symmetry in AA-stacked layers, which fosters a higher second-order nonlinear susceptibility, especially evident in crystals synthesized through atmospheric-pressure chemical vapor deposition (APCVD). Figure 6(a) illustrates the FWM and SHG processes in these stacked MoS_2_ crystals, while Figure 6(b) shows a power dependence analysis, confirming the quadratic behavior of SHG intensity with respect to the laser power, a hallmark of nonlinear optical effects [126].

Notably, the stacking-dependent nonlinear optical response extends beyond second-order processes. For instance, SHG is predominantly observed in odd-numbered layers of AB-stacked MoS_2_ due to the absence of centrosymmetry in these configurations. On the contrary, even-layered structures exhibit a minimal SHG response due to the cancellation of nonlinear dipoles as mentioned in Figure 2. The variation in SHG intensity across different stacking orders, as shown in Figure 6(c), underscores the complex interplay between layer arrangement and nonlinear optical activity [126]. FWM exhibits a quadratic increase with the number of 2D material layers. This suggests that the signal source is the nonlinear optical susceptibility not the symmetrical element, which is related to the number of dipoles present.

In addition to SHG, TMDs such as MoS_2_, WS_2_, and ZrTe_2_ demonstrate intricate nonlinear absorption dynamics. Investigations using optical *Z*-scan and photoacoustic *Z*-scan techniques reveal a mixture of saturable absorption (SA) and reverse saturable absorption (RSA) in these materials, which contributes to their tunable third-order nonlinearities. Figure 6(d) provides insights into the nonlinear absorption behavior, contrasting the nonlinear scattering and absorption dynamics in TMDs, while the inset presents the corresponding absorption coefficients and saturation intensities. These variations in nonlinear response are crucial for applications like optical limiting and switching [127].

TMDs are also known to exhibit strong nonlinear effects under conditions of broken symmetry. In spiral WS_2_ nanosheets, for instance, the twisted screw-like atomic structure induces significant nonlinear optical responses. As Figure 6(e) and (f) illustrates, SHG and THG intensities increase with layer thickness in these spiral structures, deviating from the expected diminishing SHG oscillations observed in conventionally stacked TMDs [128]. The increased nonlinear susceptibility arises from the unique AA stacking order in these spiral nanosheets, as confirmed by aberration-corrected transmission electron microscopy (AC-TEM). This broken symmetry enables not only enhanced second-order processes but also third-order nonlinear phenomena, making spiral WS_2_ a promising candidate for high-efficiency nonlinear optical applications. Further, the dependence of SHG on layer thickness and stacking configurations extends to other TMD materials. It is noteworthy that the sum frequency generation (SFG) intensity of the 3-layered system surpasses that of the 5-layered system. This observation suggests the presence of an additional electronic resonance effect that contributes to SFG signal generation when an extra excitation beam is introduced [132]. Figure 6(g) illustrates how SHG and THG intensities in TMDs vary with layer thickness, showing that the nonlinear response can be tailored by adjusting the layer count – an essential feature for designing TMD-based devices for wavelength conversion and frequency doubling [129]. Figure 6(h) presents a three-dimensional surface profile of stacked TMDs, highlighting structural variations that affect nonlinear optical performance. The morphology and layer continuity directly impact SHG and THG efficiency, underscoring the importance of precise structural control. Figure 6(i) compares THG and SHG spectra in WS_2_ and MoS_2_, revealing that tailored bandgaps and electronic structures enable efficient higher-order harmonic generation. This versatility supports TMDs’ role in nonlinear optics, where multiple harmonic frequencies can be generated from a single source [130]. Lastly, Figure 6(j) shows polarization-dependent SHG patterns in WS_2_ and MoS_2_, demonstrating their anisotropic response. This directional SHG variation, based on the polarization angle, is advantageous for applications in nonlinear optical switches and polarization-sensitive detectors [131].

## 5 Graphene

Graphene, renowned for its massless Dirac fermions and gapless electronic band structure, exhibits remarkable nonlinear optical behavior despite its centrosymmetric nature. While second-order nonlinearities are absent in this material, it exhibits a range of higher-order nonlinear optical responses. These include the dominant THG and FWM, as well as the recently observed Six- and Eight-Wave Mixing [133]. These processes have been extensively studied in recent years, particularly in relation to gate-tunable properties and strain engineering techniques that further enhance graphene’s nonlinear susceptibilities [134], [135], [136].

**Figure 7:**
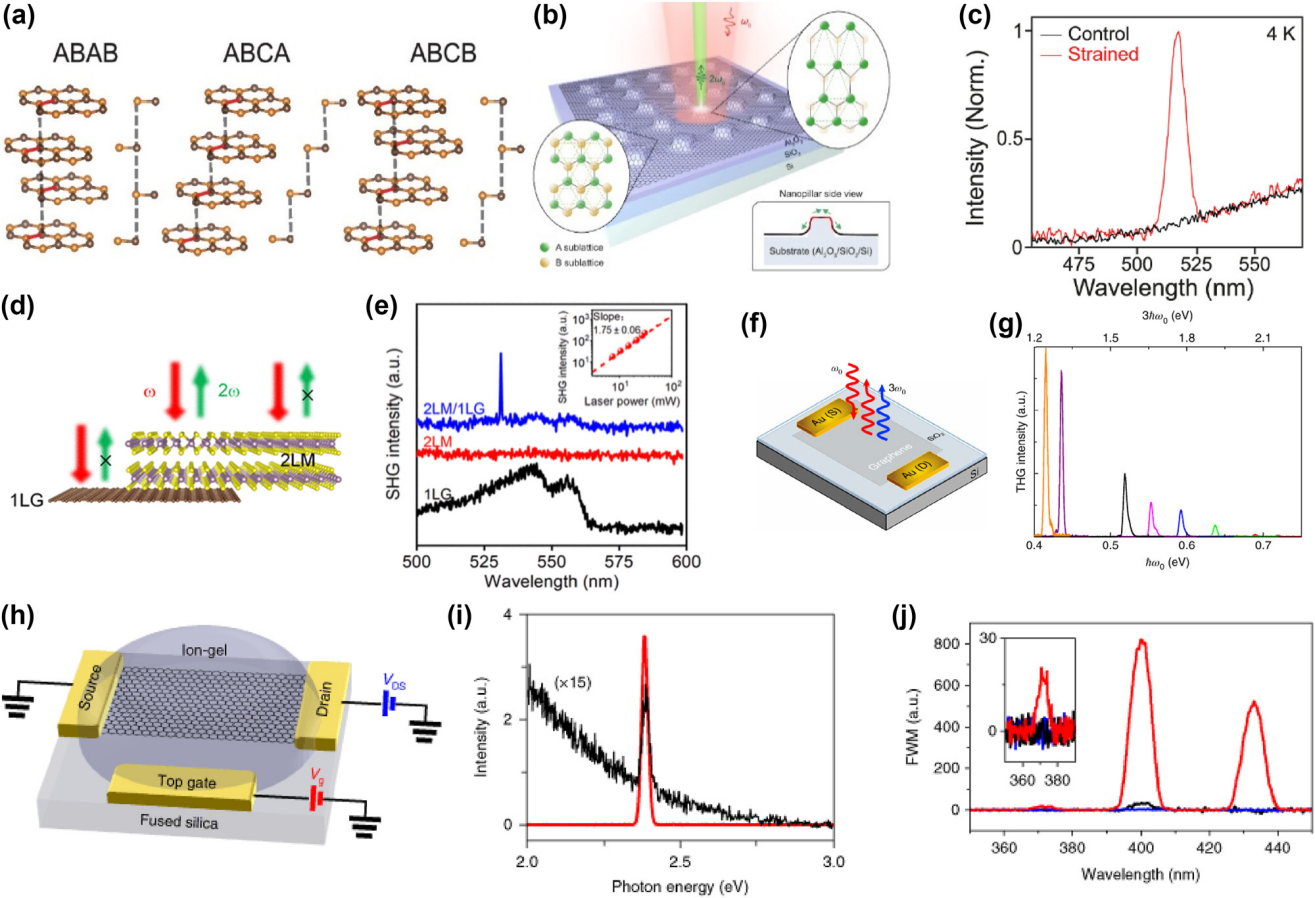
Nonlinear optical phenomena in graphene, highlighting SHG, THG, and FWM enhancement via strain, heterostructures, and gating. (a) Various arrangements of graphene tetralayers with ABAB, ABCA, and ABCB stacking orders enhancing SHG [137]. Reprinted with permission Copyright 2024 American Chemical Society. (b) Schematic illustration of strained graphene sample, which enables SHG near the edge of the nanopillars upon the excitation of ultrafast lasers. (c) Emission spectra of pristine (black) and strained (red) graphene measured with strained graphene showing a very sharp SHG peak at half of the pump wavelength [138]. Reproduced with permission under Creative Commons license. (d) Schematic of the emergent SHG in graphene/molybdenum disulfide heterostructure. (e) Frequency-up-converted optical spectra measured with SHG enhancement exhibited graphene/molybdenum disulfide heterostructure [139]. Reproduced with permission under Creative Commons license. (f) Schematic of exfoliated graphene on Si/SiO for third harmonic generation (THG) (g) representative third harmonic spectra for different incident *ħω*_0_ for SLG on sapphire [140]. Reproduced with permission Copyright 2018 Springer Nature. (h) Schematic of an ion-gel-gated graphene monolayer on a fused silica substrate covered for enhanced third-harmonic generation (i) measured THG spectra by a normally incident femtosecond input pulse at 1,566 nm from graphene gated at *μ* = 0 (black curve, magnified by 15 times) and *μ* = −0.74 eV (red curve). (j) Output spectra of FWM of the ion-gel gate graphene [141]. Reproduced with permission Copyright 2018 Springer Nature.

Although graphene’s centrosymmetry prohibits intrinsic second-order effects, the introduction of strain can break this symmetry and enable SHG. Figure 7(a) illustrates various stacking configurations of tetralayer graphene, where the ABCB stacking configuration breaks inversion symmetry, allowing for pronounced SHG. In contrast, the ABAB and ABCA configurations retain inversion symmetry and show little to no SHG response. The broken inversion symmetry in ABCB-stacked graphene introduces ferroelectric-like properties, leading to significant SHG intensities [137].

Similarly, strain engineering in graphene can activate SHG by breaking the sublattice symmetry. Figure 7(b) provides a schematic of strained graphene on nanopillars, where localized strain fields generate SHG near the edges of the structure. Figure 7(c) shows the emission spectra of pristine and strained graphene, highlighting the sharp SHG peak in strained samples due to sublattice polarization and pseudo-Landau levels [138]. Beyond second-order processes, graphene exhibits strong third-order nonlinear effects, including THG. Gate-tunable THG in graphene offers a versatile platform for optical frequency conversion and signal processing, as it allows the modulation of THG efficiency across a broad range of frequencies. Figure 7(d) illustrates a schematic of a graphene/molybdenum disulfide heterostructure, which exhibits enhanced SHG due to interlayer coupling. This van der Waals heterostructure introduces centrosymmetry breaking, enabling strong SHG in otherwise centrosymmetric materials.

Furthermore, Figure 7(e) depicts the frequency-up-converted optical spectrum from the SHG-enhanced graphene/molybdenum disulfide heterostructure, where harmonic peaks are clearly visible. This demonstrates the potential for hybrid structures to amplify nonlinear optical responses through interlayer interactions [139]. Figure 7(f) presents a schematic of ion-gel-gated graphene, which enables gate-controlled tuning of THG. The gating modulates graphene’s chemical potential, allowing the THG response to be adjusted with precision. Gate-tuning is a powerful method to control the Fermi level, as illustrated by the sharp changes in THG efficiency at different gate voltages. Graphene’s tunable third-order nonlinearity, particularly in THG, spans a wide frequency range, making it suitable for broadband applications. Figure 7(g) shows representative THG spectra for various photon energies, highlighting how THG intensity increases as the gate voltage is tuned. The tunability is particularly pronounced near resonant conditions, where the THG efficiency is dramatically enhanced, opening pathways for dynamic control in nonlinear optical devices [140].

Figure 7(h) highlights the measured THG spectra at different chemical potentials, showing significant modulation of THG intensity based on the gate voltage. This effect is due to the resonant enhancement of multiphoton transitions, demonstrating the versatility of graphene in tunable nonlinear photonic applications. In addition to THG, graphene exhibits strong FWM capabilities. Figure 7(i) demonstrates the output spectra of FWM in ion-gel-gated graphene. Gate-tunable FWM in graphene allows precise control over the nonlinear interaction, as shown by the modulation of FWM output with gate voltage. This feature makes graphene a promising candidate for optical multiplexing, switching, and frequency conversion technologies. Moreover, Figure 7(j) shows the gate-tunable third-order nonlinear optical response in graphene. As the gate voltage is adjusted, the Fermi energy of graphene shifts, modulating its nonlinear susceptibility [141]. This tunability allows control over processes like Self-Phase Modulation (SPM) and Cross-Phase Modulation (XPM), expanding the possibilities for graphene-based nonlinear photonic devices.

## 6 Black phosphorus (BP)

Black phosphorus (BP) has garnered significant interest due to its unique anisotropic band structure and tunable optical properties, making it a compelling material for both linear and nonlinear photonic applications. Its layer-dependent bandgap, which ranges from 0.3 eV in bulk to 2 eV in a monolayer, positions BP as an exceptional candidate for mid-infrared (mid-IR) to near-infrared (near-IR) applications. In terms of nonlinear optics, BP exhibits primarily third-order nonlinearities, including THG, given its centrosymmetric crystalline structure. However, recent developments have demonstrated that SHG can be achieved in BP under specific conditions, particularly through symmetry breaking caused by environmental factors like air exposure [142].

**Figure 8:**
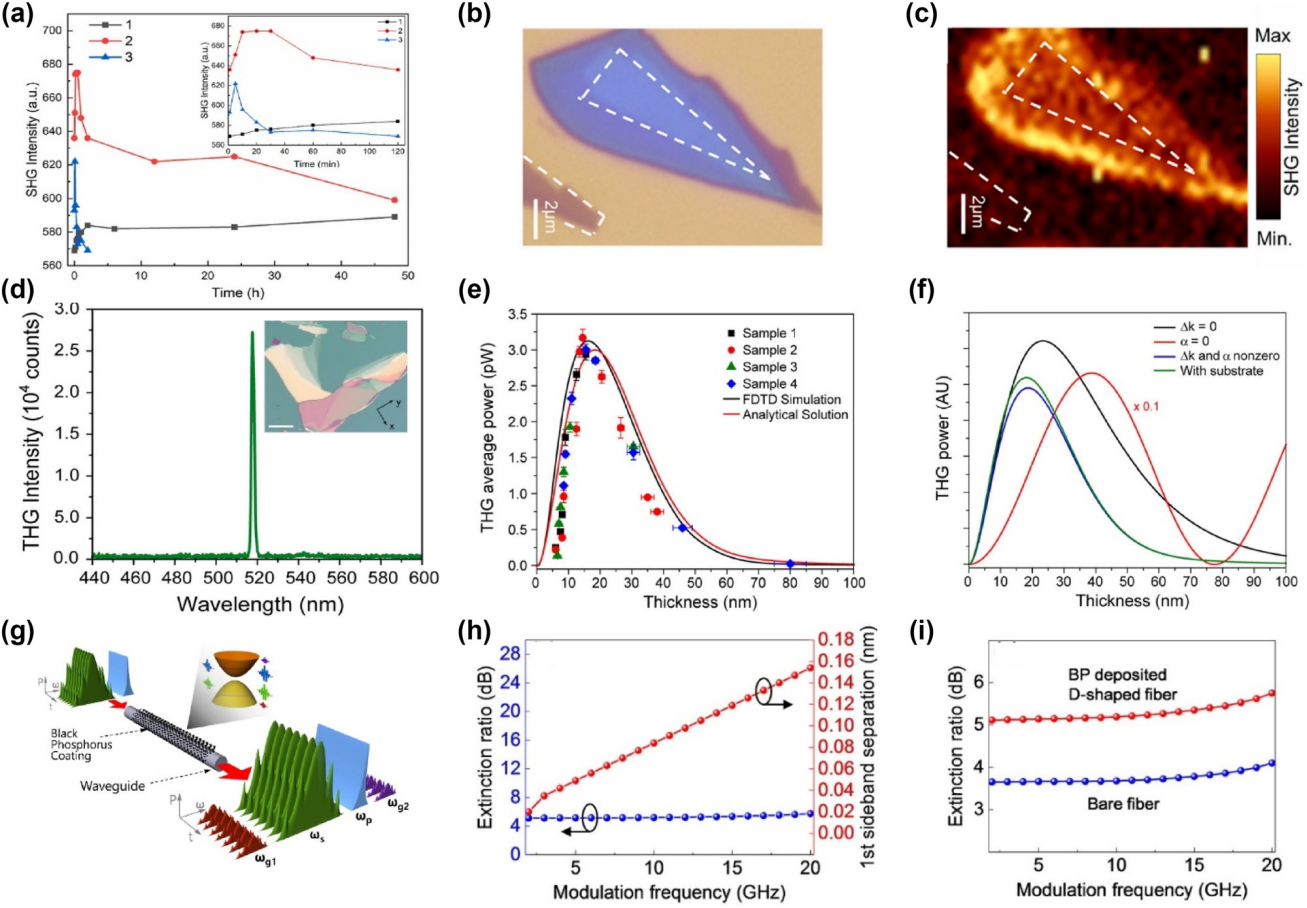
Nonlinear optical phenomena in black phosphorus (BP), focusing on SHG, THG, and FWM, with insights into thickness, edge effects, and substrate interactions. (a) Time-dependent of SHG intensity of (black phosphorous) BP flakes. (b, c) Optical and SHG image of BP flakes with different areas divided by white dotted lines. The SHG emission in the edge area is stronger than that in the middle area. The thinnest BP flakes at the corner have no SHG emission [143]. Reproduced with permission Copyright 2022 Elsevier. (d) Third-harmonic generation (THG) in multilayer BP with a peak wavelength at 519 nm, which is 3 times the frequency of the fundamental excitation. Inset is optical image of BP flake with multiple layers visible (e) THG average power plotted versus thickness. The solid black line corresponds to full FDTD simulation, where *χ* ^(3)^ was assumed to be constant and the thickness of the BP layer was varied on a 300 nm SiO_2_/Si substrate. (f) Analytical model of THG in BP showing the contributions of optical absorption, phase mismatch, and reflection of THG from the substrate [144]. Reproduced with permission Copyright 2017 American Chemical Society. (g) Schematic illustration of FWM-based wavelength conversion in BP-deposited nonlinear fiber optic device. (h) Separation of the first sideband of the newly generated signal. The red line is distance from the main peaks to first sidebands, and blue line is for the extinction ratio per modulation frequency. (i) Efficiency comparison of the generated signals without and with the BP-deposited fiber [145]. Reproduced with permission under Creative Commons license.

One of the most exciting aspects of BP is its layer-dependent third-harmonic generation. Due to its tunable bandgap and anisotropic optical response, BP’s nonlinear optical properties, including THG, can be finely controlled by altering the number of layers. Figure 8(a) shows an optical image of a multilayer BP flake, with multiple layers clearly visible, and Figure 8(b) illustrates the corresponding THG emission captured by a CCD camera, confirming that the THG signal emanates from the flake’s surface. The THG spectrum, shown in Figure 8(c), exhibits a strong peak at 519 nm, which is precisely one-third of the fundamental wavelength (1,557 nm), confirming the occurrence of third-harmonic generation [143]. The power dependence of the THG signal is presented in Figure 8(d), where a cubic power law relationship is observed, further validating the third-order nonlinear response. BP’s nonlinear optical properties are highly sensitive to both thickness and polarization of the incident light. The THG power varies significantly with the number of layers due to the interplay between phase-matching conditions and absorption. Figure 8(e) demonstrates the THG power as a function of BP thickness, where an optimal thickness for maximum THG output is observed. As the thickness increases beyond this point, the THG signal diminishes due to absorption and depletion effects. Moreover, BP exhibits strong anisotropy in its THG response. The dependence of THG on the incident polarization is shown in Figure 8(f), where the maximum THG signal is obtained when the pump polarization aligns with the armchair direction of the BP crystal, and the signal is minimized along the zigzag direction [144]. This anisotropic behavior arises from the intrinsic crystal structure of BP, where the effective mass of carriers differs significantly between the armchair and zigzag directions, leading to directionally dependent nonlinear optical responses.

While BP is inherently centrosymmetric, and thus SHG is theoretically forbidden, recent studies have shown that SHG can be induced by exposing BP to air, which breaks its inversion symmetry through oxidation. Figure 8(g) depicts a BP flake after air exposure, where oxidation creates oxygen defects that disrupt the symmetry, enabling SHG. The corresponding SHG signal is shown in Figure 8(h), where a clear peak at 532 nm, corresponding to SHG emission, is observed. Air-exposure time and BP flake thickness play critical roles in modulating SHG intensity. Thinner BP flakes degrade more rapidly upon exposure to air, which accelerates the oxidation process and leads to faster changes in SHG intensity. As shown in Figure 8(i), the SHG intensity evolves over time for different BP flake thicknesses. Initially, SHG intensity increases as oxidation progresses but eventually decreases as severe degradation destroys the periodic lattice structure [145].

## 7 Nanowires

Nanowires represent a unique class of low-dimensional materials, where quantum confinement effects and surface-to-volume ratios significantly influence their nonlinear optical (NLO) properties [146], [147]. Nanowires, due to their anisotropic structures, exhibit intriguing nonlinear behavior such as SHG and THG, with potential applications in photonics, sensing, and nanodevice technologies. These effects can be further enhanced or modulated by structural design and external stimuli, such as electric fields, offering a versatile platform for nonlinear optical investigations.

**Figure 9:**
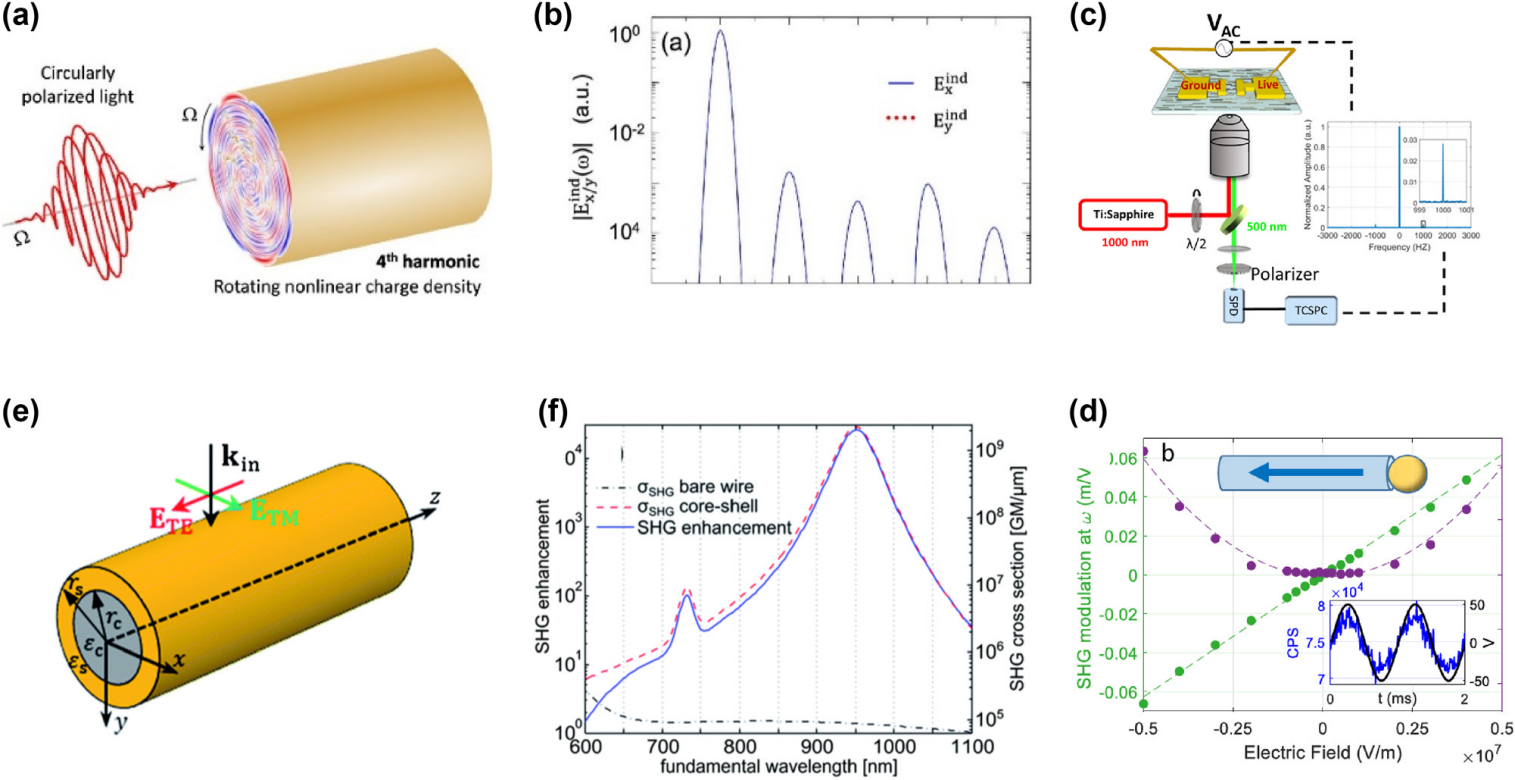
Nonlinear optical phenomena in plasmonic nanowires, emphasizing SHG modulation under external fields and enhancement in core-shell nanostructures. (a) Schematic of plamsonic nanowire with intense circularly polarized electromagnetic external field pulse for NLO (b) spectral analysis of nonlinear near fields and multipole moments [148]. Reproduced with permission under Creative Commons license. (c) Measurement of NLO of a nanowire between two electrodes using an inverted microscope. The SHG signal at 500 nm is collected by a single-photon detector (SPD) and time-stamped with a time-correlated single-photon counting (TCSPC) system. (d) SHG modulation against the external applied field strength for a right-growing nanowire [149]. Reproduced with permission under Creative Commons license. (e) Simulation of the optical properties of KNbO_3_ nanowires considered geometry. (f) SHG enhancement of a core–shell Au-KNbO_3_ [150]. Reproduced with permission Copyright 2014 Royal Society of Chemistry.

The modulation of the nonlinear optical response in nanowires under an external electric field has been a subject of recent research, particularly in polar materials like ZnO. Figure 9(a) illustrates the concept of electric-field-induced SHG in ZnO nanowires, where a rotating nonlinear charge density under circularly polarized light leads to fourth harmonic generation. The electric field plays a key role in this modulation, as it interacts with the polar nature of the nanowires, leading to variations in SHG intensity. Figure 9(b) presents the electric field’s effect on SHG intensity as a function of the applied electric field strength. When the field is applied parallel or anti-parallel to the polar axis, SHG intensity increases or decreases, respectively, demonstrating the tunability of nonlinear optical properties in polar nanowires [148].

Polar materials like ZnO exhibit distinct nonlinear properties due to their inherent electric dipoles. The polarity of these nanowires can be exploited to modulate nonlinear optical effects, as illustrated in Figure 9(c), where the electric field’s interaction with a polar ZnO nanowire alters SHG intensity. This external-field-induced SHG (EFISH) effect allows the determination of the nanowire’s polarity and is a powerful tool for material characterization at the nanoscale. Furthermore, as shown in Figure 9(d), the SHG modulation as a function of electric field strength highlights the interference between static SHG and EFISH, providing insights into the second-order nonlinear susceptibilities of the material. This behavior is crucial for applications in optoelectronic devices where electric fields can be used to tune the optical properties of nanostructures [149].

In addition to external fields, structural modifications such as the introduction of a plasmonic shell around nanowires can dramatically enhance their nonlinear response. Core–shell nanowires, where the core material exhibits intrinsic nonlinear properties and the shell provides plasmonic enhancement, have shown remarkable promise in increasing SHG output. Figure 9(e) shows the design of core–shell potassium niobate (KNbO_3_) nanowires, where the plasmonic resonance of the gold shell boosts the SHG signal of the nonlinear KNbO_2_ core. The corresponding enhancement in SHG is depicted in Figure 9(f), where SHG intensity from bare nanowires is compared to that of core–shell nanowires. The core–shell configuration leads to a significant enhancement in SHG across a broad wavelength range, particularly around the plasmon resonance wavelength of the gold shell. This plasmon-enhanced nonlinear effect opens up avenues for integrating nanowires into photonic circuits and sensing devices [150].

## 8 Quantum dots (QDs)

**Figure 10:**
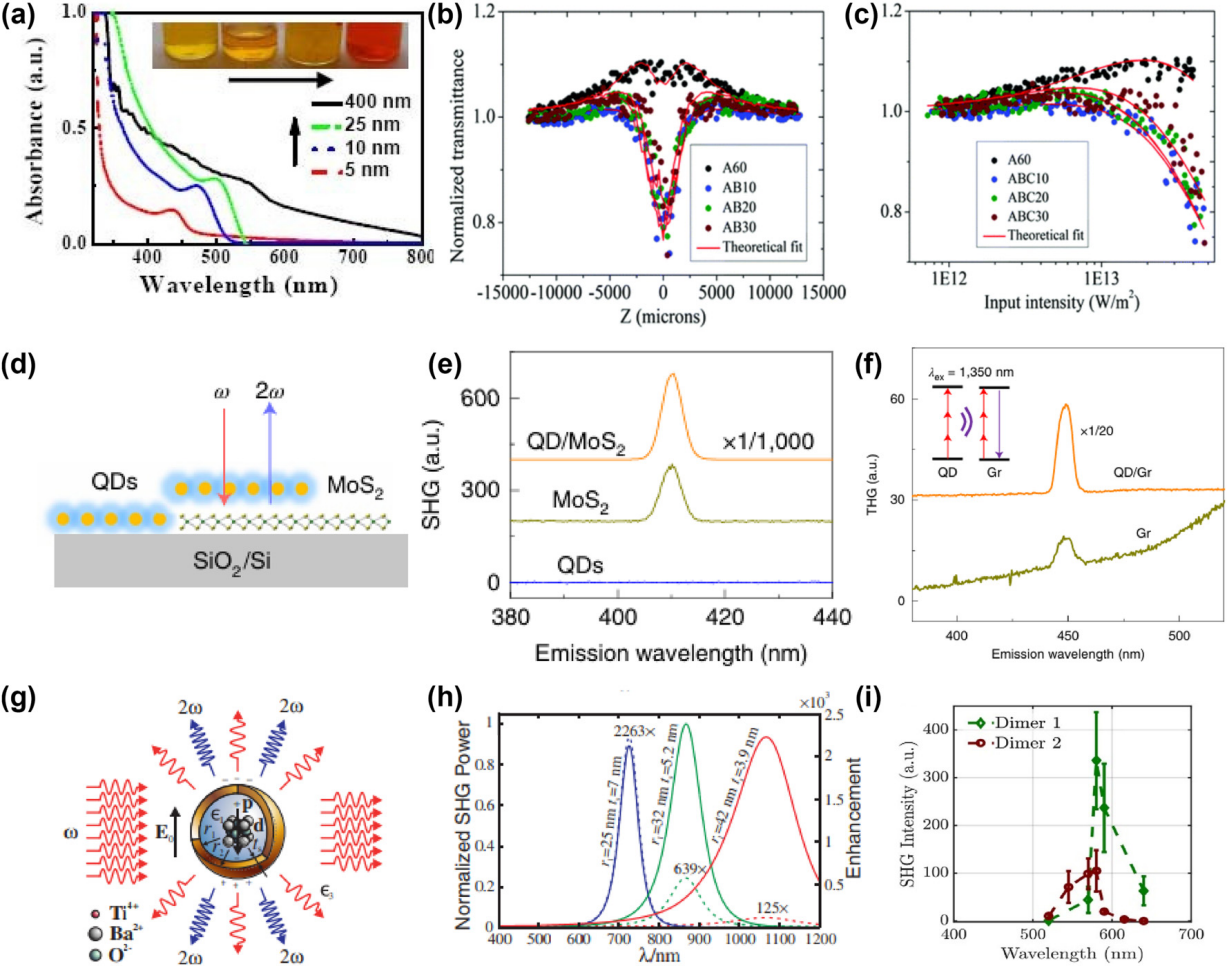
Nonlinear optical phenomena in quantum dots (QDs) and hybrid nanostructures, showcasing enhanced SHG and THG in QD/MoS2 hybrids, plasmonic nanocavities, and BaTiO3 nanodimers. (a) Optical absorption spectra of CdSe nanoparticles of different sizes [153]. Reproduced with permission under Creative Commons license. (b) Open aperture *Z*-scan curves and (c) normalized optical transmission curves obtained from the *Z*-scan curves for CdSe/CdS/ZnS QDs of various shell thickness [154]. Reproduced with permission Copyright 2019 Royal society of chemistry. (d) Schematic of the QD/MoS_2_ hybrid structure and its enhanced SHG (e) SHG spectra of QD/MoS_2_ under 820 nm pulsed-laser excitation boosted by ∼1,500 times in the hybrid. (f) Enhancement of third harmonic generations after QD coating under excitation at 1,350 nm. Enhancement and tunability of plasmonic SHG nanocavities [155]. Reproduced with permission Copyright 2021 Springer Nature Limited. (g, h) Principle and varied plasmonic SHG nanocavities. Solid curves represent radiation power in second-harmonic frequency normalized to the maximum radiation power among the three examples. Dotted curves represent the factor of SHG enhancement compared to the core [156]. Reproduced with permission Copyright 2010 American Physical Society. (i) Wavelength dependence SHG from two distinct BaTiO_3_ nanodimers [157]. Reproduced with permission Copyright 2017 American Chemical Society.

Quantum dots (QDs), as zero-dimensional semiconductor materials, exhibit unique quantum confinement effects that enhance their nonlinear optical properties, such as multiphoton absorption and SHG. These phenomena arise from the discrete energy levels of QDs, enabling interactions with multiple photons simultaneously and opening applications in imaging, sensing, and photonic devices [151], [152]. The nonlinear optical behavior of QDs is highly size-dependent. CdSe quantum dots, in particular, have been studied extensively for their size-dependent multiphoton absorption and refraction properties. Figure 10(a) shows the absorbance spectra of CdSe QDs of various sizes. As the size decreases from 400 nm to 5 nm, the absorbance edge shifts toward the blue, illustrating the strong quantum confinement effects in smaller QDs [153].

In terms of nonlinear optical phenomena, smaller QDs (under 10 nm) display four-photon absorption (4PA), whereas larger QDs (25 nm and 400 nm) show three-photon absorption (3PA). This distinction is due to the relationship between QD size and the Bohr exciton diameter, which influences quantum confinement and, in turn, multiphoton absorption characteristics. Figure 10(b) presents *Z*-scan results for CdSe QDs, highlighting how transmittance changes with input intensity, further affirming the size-dependent nonlinear behavior of these nanoparticles. To enhance nonlinear absorption further, core–shell architectures are widely employed. In CdSe/ZnS core–shell QDs, for example, the ZnS shell modifies the local field around the core, boosting the nonlinear absorption properties. Figure 10(c) illustrates this core–shell structure, where the surrounding shell increases the nonlinear absorption cross-section through dielectric confinement [154]. The SHG spectra comparison in Figure 10(d) between core–shell systems and bare CdSe QDs demonstrates the considerable enhancement achieved with this architecture. Beyond individual QD structures, hybrid systems combining QDs and two-dimensional materials have been shown to amplify SHG significantly. When CdSe QDs are paired with a MoS_2_ monolayer, the SHG signal is intensified through multiphoton-excitation resonance energy transfer (MPERT) from the QDs to the MoS_2_. Figure 10(e) shows this hybrid system, where QDs deposited on MoS_2_ enhance the SHG response through dipole–dipole coupling. Figure 10(f) presents the SHG spectra of MoS_2_, CdSe QDs, and the QD/MoS_2_ hybrid system. While MoS_2_ alone has a weak SHG response, integrating CdSe QDs amplifies the SHG intensity by more than 1,500 times. This enhancement is due to efficient energy transfer from the QDs to MoS_2_, greatly boosting the nonlinear response of the system [155].

Core–shell nanodimers represent another structure that significantly enhances SHG. For instance, barium titanate (BaTiO_2_) nanoparticles coated with a gold shell exhibit strong SHG due to the plasmonic resonance in the gold shell. Figure 10(g) depicts this core–shell nanodimer, where the gold shell acts as a nanoantenna, concentrating the electric field and intensifying the SHG from the BaTiO_2_ core. The impact of these core–shell structures on SHG is further illustrated in Figure 10(h), which shows SHG enhancement for different core–shell combinations. The localized surface plasmon resonance (LSPR) effect in the gold shell boosts SHG efficiency by several orders of magnitude, underscoring the power of plasmonic materials in enhancing nonlinear optical effects [156]. Lastly, Figure 10(i) demonstrates the benefits of assembling hybrid nanoparticle structures for SHG enhancement. In this configuration, CdSe QDs are arranged as dimers with plasmonic particles, creating a collective response that greatly increases SHG intensity. This dimer structure shows significantly higher SHG than individual QDs, highlighting the advantages of particle assembly in augmenting nonlinear optical responses [157].

## 9 Applications and current progress

### 9.1 Laser technology and fiber lasers

**Figure 11:**
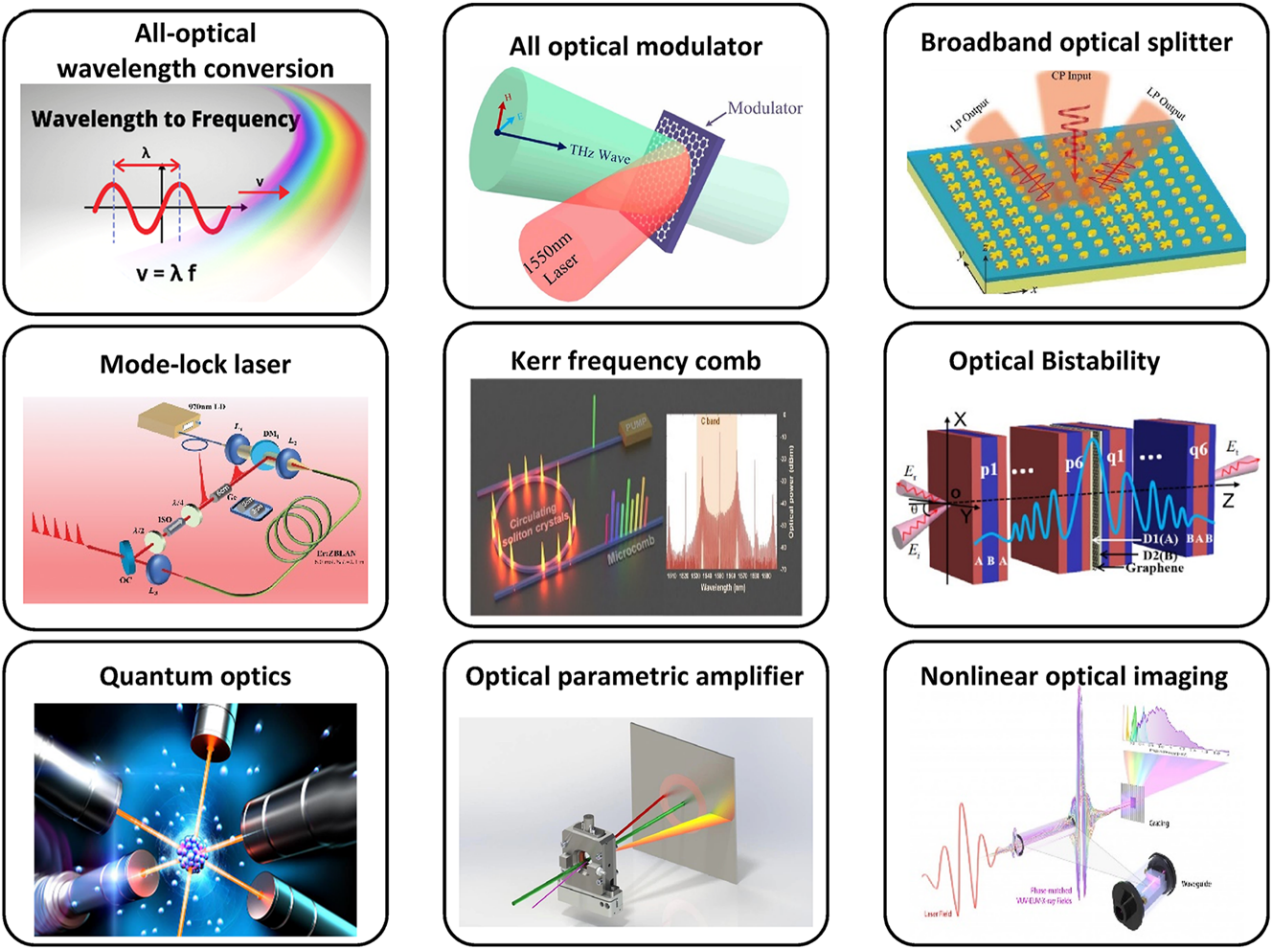
Common applications of nonlinear optical processes.

Nonlinear optics in low-dimensional materials have found significant applications in laser technology and fiber lasers. One of the key applications of these materials in laser technology is in the development of fiber lasers with saturable absorbers, a property that is crucial for the generation of ultrafast light pulses in mode-locked lasers [116], [158]. Low-dimensional materials have been shown to exhibit excellent saturable absorption properties, making them ideal for use in fiber lasers. TMDs are widely used in nonlinear frequency conversion systems, including SHG and THG. Their non-centrosymmetric crystal structures and tunable bandgaps make them efficient platforms for frequency doubling and wavelength mixing in laser systems. For instance, recent advances have been made in vector soliton fiber lasers using low-dimensional materials as saturable absorbers [159], [160]. In fiber lasers, solitons can be used to generate ultrafast light pulses, and low-dimensional materials have been shown to enhance the generation of these solitons. Moreover, low-dimensional materials have been used to improve the performance of ultrafast fiber lasers [161]. Notably, mixing beams in the reverse direction within a fiber optic laser presents intriguing possibilities, especially when incorporating low-dimensional materials. Counter-propagating beams intensify light–matter interaction, leading to stronger nonlinear optical effects, particularly in low-dimensional materials [162]. This can be exploited to generate new frequencies, optical switching, and advanced optical sensing applications. However, overcoming challenges such as phase-matching, loss management, and material selection is crucial for realizing practical implementations (Figure 11).

## 10 Ultrafast photonics and high-speed optical communications

Recent research has focused on the utilization of low-dimensional materials for ultrafast photonics, which involves the generation, manipulation, and detection of light pulses on extremely short timescales [163]. Graphene-based optical switches have emerged as key enablers in ultrafast photonics, exploiting their exceptionally high third-order nonlinearities to achieve femtosecond-level modulation and optical switching. These devices are pivotal for high-speed optical communication networks and frequency comb generation. Their unique nonlinear optical properties are particularly relevant for applications such as signal processing, all-optical switching, and the development of high-speed photonic devices [164]. The ultrafast response speed of these materials makes them well-suited for high-speed optical communications, offering opportunities to improve the efficiency and speed of optical data transmission [165], [166].

## 11 Integrated photonics devices

In the field of nonlinear integrated photonics, the exploitation of nonlinear optical effects within photonics structures with dimensions comparable to or much smaller than the wavelength of light has led to the development of groundbreaking new devices and applications [167]. These materials are particularly well-suited for use in integrated photonic devices due to their high nonlinear optical coefficients and ultrafast response speeds, which are essential for achieving efficient and compact photonic devices [37]. The development of nonlinear integrated photonics (NLIP) has greatly expanded due to the development of novel light advanced materials and very effective guiding structures. Waveguide geometries, in particular, offer the best prospects for optimizing the efficiency of nonlinear devices, making them an ideal platform for the integration of low-dimensional materials [42]. Low-dimensional heterostructures, such as graphene-TMD hybrids, further enhance the capabilities of integrated photonic devices by combining complementary nonlinear properties. These systems enable tunable frequency conversion, all-optical modulation, and broadband wavelength control in compact configurations [33], [161]. The materials of interest for realizing nonlinear optical devices are typically in bulk form. However, the combination of integrated optics technologies with nonlinear photonics has opened the way to the use of low-dimensional materials in integrated photonic devices. These materials have become of paramount importance for their dual role as sources of detrimental effects and as key components for the development of advanced integrated photonic devices. The unique nonlinear optical properties of these materials make them indispensable for achieving efficient and compact photonic devices, ultimately leading to the development of more advanced and versatile integrated photonic systems [64], [168].

## 12 Nonlinear light control and signal processing

In the realm of nonlinear light control, researchers have harnessed low-dimensional materials to achieve both efficiency and versatility in manipulating light properties. Their high nonlinear optical coefficients and ultrafast response speeds make them ideal candidates for applications such as all-optical switching, wavelength conversion, and the generation of ultrafast light pulses [169]. These materials offer the potential to develop compact and efficient nonlinear light control devices, which are crucial for various photonic applications. Furthermore, the application of low-dimensional materials in signal processing has shown significant promise. Their unique nonlinear optical properties enable the manipulation and processing of optical signals with high efficiency and speed. This includes applications such as signal regeneration, wavelength division multiplexing, and the development of high-speed photonic devices [164]. The ultrafast response speed of these materials makes them well-suited for high-speed signal processing, offering opportunities to improve the efficiency and speed of optical communication systems [170], [171].

## 13 Bio-imaging and medical applications

Low-dimensional materials find significant application in bio-imaging, leveraging their nonlinear optical properties to achieve high-resolution imaging of biological samples [172]. For instance, these materials have been employed in two-photon excited fluorescence imaging of vasculature and neurons *in vivo*, SHG imaging of collagen tissues, THG microscopic imaging of tissues *in vivo*, coherent anti-Stokes Raman scattering imaging of tissues with CH_2_ contrast, stimulated Raman scattering imaging of biological samples, and transient absorption imaging of heme granule dynamics in *C. elegans* [31]. Quantum dots, with their discrete energy levels and size-tunable nonlinear optical properties, are playing a transformative role in bioimaging. They enable enhanced resolution and multiphoton excitation for real-time imaging in complex biological environments, paving the way for advanced diagnostics and targeted imaging. Importantly, all non-linear optical signals generated within the molecular system, or the surrounding biological media, are achieved through the critical process of phase-matching [173]. The unique nonlinear optical properties of low-dimensional materials also make them promising for medical applications. Specifically, their capacity for label-free and chemical-specific imaging holds significant promise in the fields of medical diagnostics and research [174]. Additionally, the development of advanced nonlinear optical materials and devices based on low-dimensional materials holds promise for enhancing various medical applications, including bio-imaging, material structure analysis, and optical signal processing.

## 14 Challenges and perspectives

The field of nonlinear optics in low-dimensional materials faces several current challenges. One significant challenge is the need to engineer the nonlinear optical properties of 2D materials, including graphene, TMDs, and BP. While these materials show promise due to their atomic thickness and tunable light–matter interactions, achieving the desired nonlinear optical responses requires further research and development. Additionally, understanding and optimizing the material parameters that influence nonlinear optical properties, particularly third-order nonlinearities, remain areas of active investigation.

While these challenges have been widely acknowledged, addressing them requires innovative approaches in synthesis, device design, and integration. Key challenges in the field of nonlinear optics with low-dimensional materials include developing scalable and reproducible synthesis techniques for high-quality materials. Methods such as CVD and atomic layer deposition (ALD) must be optimized for cost-effectiveness and large-scale production. Additionally, phase-matching techniques tailored to low-dimensional systems require innovation. Approaches such as twist-angle engineering in layered 2D materials and hybrid integration with metasurfaces have demonstrated potential in overcoming conventional phase mismatch. Overcoming the challenge of limited interaction lengths in these materials through the integration with photonic resonators such as Fabry–Pérot cavities and micro-ring resonators can dramatically enhance nonlinear optical efficiencies. Such hybrid systems allow for localized field enhancement and prolonged light–matter interaction, paving the way for compact, high-performance devices.

## 15 Future directions

Looking ahead, the integration of low-dimensional materials into emerging technologies promises groundbreaking innovations. The potential solutions and future research directions to the challenges in nonlinear optics in low-dimensional materials encompass several key areas. The future of nonlinear optics in low-dimensional materials is closely tied to advancements in hybrid photonic systems and quantum technologies. Integrating low-dimensional materials with nanoscale photonic circuits offers opportunities for precise light manipulation at subwavelength scales. Research into quantum nonlinear optical phenomena, such as photon-pair generation and entanglement, has the potential to revolutionize quantum computing and secure communication networks.

Large-area material transfer and processing techniques are crucial for scalable device fabrication. Methods such as CVD and wafer-scale exfoliation enable reliable integration of low-dimensional materials into photonic devices while preserving their nonlinear optical properties. These techniques facilitate the production of devices with consistent performance across large substrates, addressing a critical need for commercial viability and real-world applications. The integration of low-dimensional materials with resonant photonic structures, such as Fabry–Pérot cavities, photonic crystals, ring resonators, and metasurfaces, represents a promising avenue for overcoming the limited interaction lengths inherent in these materials. Such hybrid systems amplify nonlinear optical processes by leveraging localized field enhancements and prolonged light–matter interactions. For instance, graphene integrated with metasurfaces has demonstrated significant enhancement in THG, enabling compact, high-efficiency photonic devices. Similarly, Fabry–Pérot cavities and ring resonators have been utilized to improve SHG and FWM efficiencies, critical for applications in ultrafast photonics and quantum computing. These hybrid structures also facilitate wavelength tunability and energy-efficient nonlinear optical responses, making them indispensable for the next generation of photonic technologies.

Moreover, scalable fabrication methods, including direct laser writing and roll-to-roll printing, are poised to make mass production of hybrid photonic systems feasible. The development of integrated quantum photonic platforms combining materials like graphene and TMDs could further unlock ultrafast signal processing and real-time sensing capabilities. These advancements will not only address existing limitations but also expand the frontiers of nonlinear optics in applications such as next-generation telecommunication and biomedical imaging.

## 16 Conclusions

In conclusion, this review paper on nonlinear optics based on low-dimensional materials presents several key findings and insights. It highlights the unique optical properties of low-dimensional (LD) materials, particularly 2D materials, which make them promising for applications in nonlinear optics. These materials offer advantages such as high chemical and mechanical stability, as well as low fabrication costs, which are desirable for practical applications. We also outline potential research directions, including the exploration of novel LD materials with enhanced nonlinear optical properties, the development of effective modulation techniques to control and enhance nonlinear optical responses, and the advancement of theoretical and experimental techniques for the characterization of nonlinear optical behaviors in these materials. Furthermore, the review emphasizes the significance of application-oriented research, showcasing the potential of LD materials in areas such as laser technology, ultrafast photonics, integrated photonics devices, and bio-imaging. Overall, the paper provides a comprehensive overview of the recent advances, theoretical and experimental aspects, and potential applications of nonlinear optics in low-dimensional materials, offering valuable insights into the future prospects of this rapidly evolving field.
